# Pulmonary hypertension in patients with Martorell hypertensive leg ulcer: a case control study

**DOI:** 10.1186/1465-9921-13-45

**Published:** 2012-06-11

**Authors:** Leonardo Glutz von Blotzheim, Felix C Tanner, Georg Noll, Matthias Brock, Manuel Fischler, Jürg Hafner, Rudolf Speich, Silvia Ulrich, Lars C Huber

**Affiliations:** 1 Pulmonary Hypertension Working Group, Clinic and Policlinic of Internal Medicine, University Hospital of Zurich, Zurich, Switzerland; 2 Department of Cardiology, University Hospital of Zurich, Zurich, Switzerland; 3 Department of Dermatology, University Hospital of Zurich, Zurich, Switzerland

**Keywords:** Pulmonary hypertension, Echocardiography, Martorell ulcer

## Abstract

**Background:**

Martorell hypertensive ischemic leg ulcer (Martorell ulcer) is characterized by distinct alterations in the arteriolar wall of subcutaneous vessels, leading to progressive narrowing of the vascular lumen and increase of vascular resistance. These changes are similar to the alterations observed in pulmonary arterioles in patients with chronic pulmonary hypertension (PH). This study was aimed to assess an association between the two disorders.

**Methods:**

In this case–control study, 14 patients with Martorell ulcer were clinically assessed for the presence of pulmonary hypertension using transthoracic Doppler echocardiography. Data from patients were compared to 28 matched hypertensive controls.

**Results:**

Systolic pulmonary arterial pressure (sPAP) in patients with Martorell ulcer was significantly higher than in the control group (33.8 ± 16.9 vs 25.3 ± 6.5 mmHg, p = 0.023); the prevalence of pulmonary hypertension was 31% (5/14) in patients and 7% (2/28) in controls (p = 0.031). No differences were seen in left heart size and function between patients and controls.

**Conclusion:**

This study provides first evidence that subcutaneous arteriolosclerosis, the hallmark of Martorell ulcer, is associated with PH. These findings suggest that patients with Martorell leg ulcer might be at significant risk to develop elevated pulmonary arterial pressure. Patients with leg ulcers who present with dyspnea should be evaluated by echocardiography for the presence of pulmonary hypertension.

## Background

Martorell hypertensive ischemic leg ulcer (HYTILU, Martorell ulcer) and pulmonary hypertension (PH) are distinct clinical entities that share common pathogenic features. Of particular interest are similar morphologic changes of arterioles that cause elevated vascular resistance. These similarities suggest a possible association between the two disorders.

**Figure 1:**
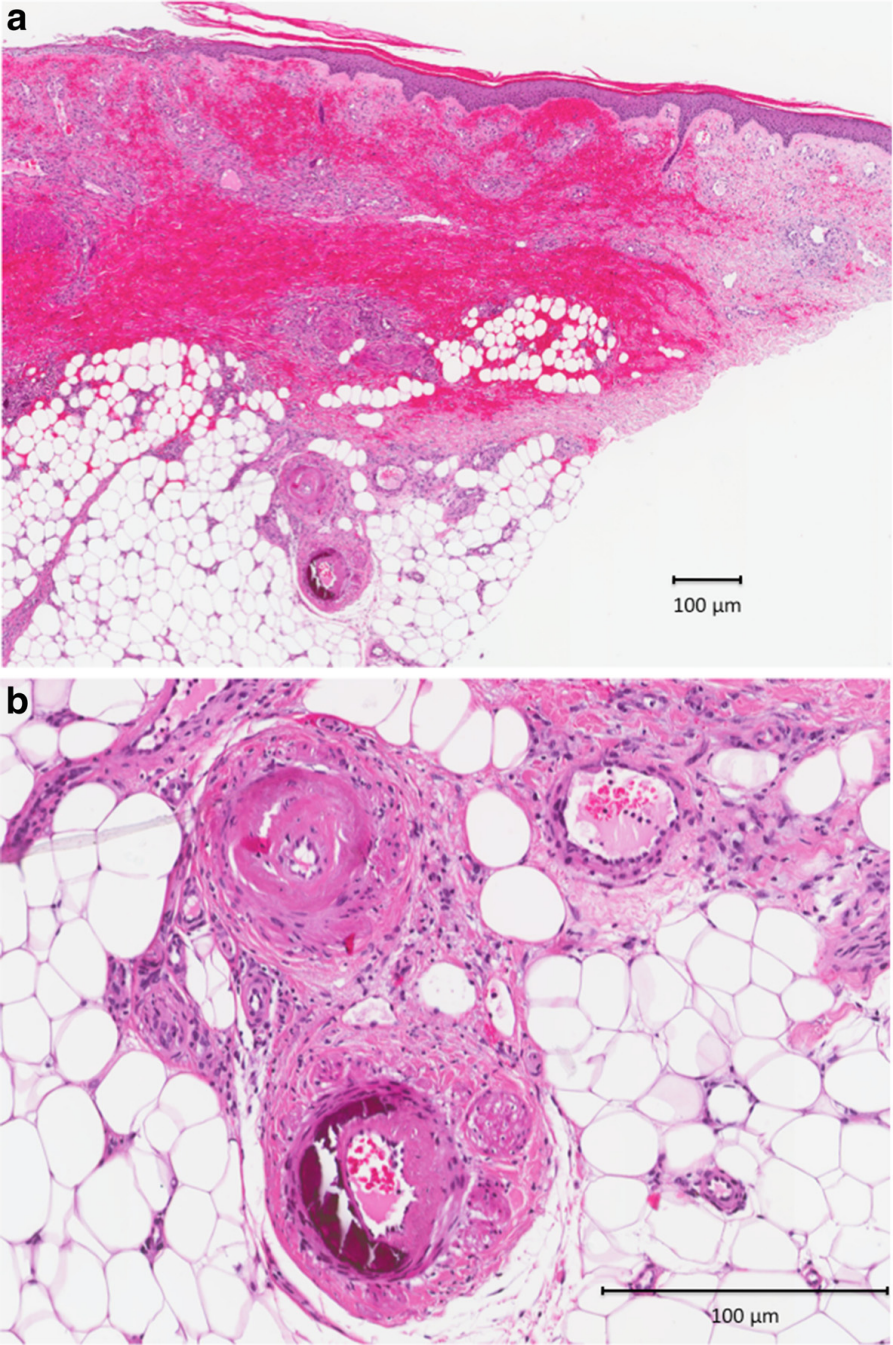
**a) Overview of necrotic skin area at the border of a Martorell hypertensive ischemic leg ulcer, containing a group of two sclerotic arterioles;** 1**b**) Higher magnification of Figure 1**a**; Group of two sclerotic arterioles, one with a thickened wall to the cost of a narrow lumen and one showing medial calcification.

In 1945, Fernandes Martorell described 4 cases of patients with ischemic leg ulcers [1] that occurred in the absence of peripheral arterial or venous disease; histological analysis of these ulcers revealed hypertensive changes of subcutaneous arterioles (i.e. hypertrophy and stenosis), which resulted in the descriptive term “hypertensive ischemic leg ulcer” [2,3]. Martorell ulcer is an entity defined by ischemic subcutaneous arteriolosclerosis of the leg in hypertensive subjects. Martorell ulcer is a rare cause for leg ulcers and is found in approximately 5% of cases. Diagnosis is based on clinical presentation, patient history and deep skin biopsy. Histology reveals hypertrophy of the smooth muscle cell layer of the vessel’s media resulting in an increased thickness of the arteriolar wall to the cost of a narrow lumen (Figure 1a/b). Systemic arterial hypertension is present in all cases, type 2 diabetes is observed in approximately 60% [4-6].

Histopathology of pulmonary vasculature in patients with P(A)H are comparable to those of Martorell ulcer. PAH is a pre-capillary disorder of the small pulmonary arteries and arterioles, characterized by extensive vasoconstriction, in situ thrombosis and vascular remodelling. Cellular changes defining the vascular remodelling include intimal fibrosis, medial and adventitial hyperplasia and a pro-proliferative, apoptosis-resistant phenotype of vascular cells. These alterations progressively narrow the arteriolar lumen and, thus, increase the pulmonary vascular resistance (PVR) leading to an increase of the right ventricular afterload and, ultimately, cardiac failure [7-10]. Of interest, these histopathological changes are not restricted to PAH but can be observed in several other forms of PH [11].

On the other hand, Martorell ulcers have been found to develop secondary to limited skin perfusion pressures resulting from an increase of vascular resistance in small cutaneous vessels. It has also been suggested that this increase of vascular resistance affects the vessel relaxation that usually follows distal to an arterial narrowing, which might further decrease tissue perfusion [12].

The striking histopathological and pathophysiologic similarities between Martorell ulcer and PAH as well as chronic PH due to other conditions prompted this case–control study. The aim was to analyse a potential association between Martorell ulcer and the development of PH.

## Methods

### Patients

**Figure 2:**
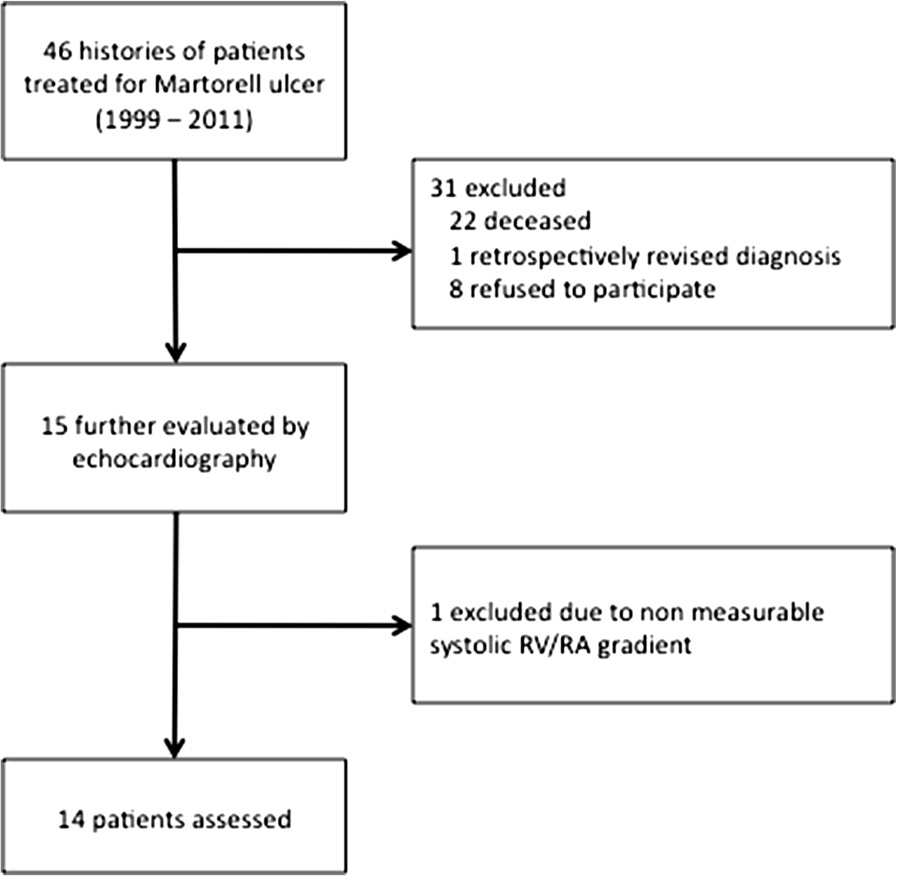
Study design.

From May 1999 to April 2011, 46 patients with Martorell ulcer were diagnosed at the Department of Dermatology, University Hospital of Zurich [4]. After reviewing the cases, a total of 15 patients were identified for further diagnostic workup according to the study design (Figure 2). Patient history was checked for i) the presence of chronic obstructive or restrictive pulmonary disease, ii) chronic thromboembolic pulmonary hypertension and iii) concomitant diagnosis of systemic disorders that have been associated with the development of pulmonary hypertension due to unclear multifactorial mechanisms (e.g. sarcoidosis, neurofibromatosis, tumoral obstruction, etc.) [13]. Patients characteristics are shown in Table 1. For each case, 2 controls were matched to gender, age at time of echocardiography (± 2 years), presence of hypertension, current smoking and presence or absence of diabetes mellitus. Data were retrieved from the electronical data pool of the hospital and included in-patients from all hospital departments. Echocardiographic workup was performed at the Department of Cardiology, University Hospital of Zurich, Switzerland (12 of 14 patients) as well as in 2 other hospitals (2 of 14 patients).

**Table 1:** Characteristics of patients and controls


Numbers investigated, n	14	28
Age at time of echocardiography – yr. (± SD)	77 (± 5)	77 (± 5)
Male sex – no. (%)	6 (42%)	12 (43%)
Present hypertension – no. (%)	14 (100%)	28 (100%)
Present diabetes – no. (%)	5 (37%)	10 (36%)
Smoker status		
*Current smoker – no. (%)*	2 (14%)	4 (14%)
*Former smoker – no. (%)*	3 (22%)	6 (21%)
*Never smoked – no. (%)*	9 (64%)	18 (64%)
Oral anticoagulation (phenprocoumon) – no. (%)	7 (50%)	8 (28%)
Antinuclear antibodies (ANA)		
*Titer ≤ 1:160 – no. (%)*	12 (86%)	n / a
*Titer > 1:160 – no. (%)*	2 (14%)	n / a

### Study design

The study was conducted at a tertiary care hospital (University Hospital of Zurich, Switzerland) and was approved by the ethics committee of the medical faculty of the University of Zurich. The study was designed as case–control study to investigate a potential correlation between the presence of Martorell ulcer and pulmonary hypertension (PH).

### Echocardiography

All patients underwent a complete transthoracic echocardiographic study. Pulmonary arterial pressure (systolic pulmonary arterial pressure, sPAP) was estimated in a non-invasive manner by measuring the maximal velocity of the tricuspid regurgitation jet and calculating the maximal instantaneous systolic pressure difference between the right ventricle and the right atrium [14]. Pulmonary hypertension was defined according to the Dana Point criteria as sPAP >36 mmHg, i.e. a tricuspid jet velocity of >2.8 m/s corresponding to an RV/RA gradient of >31 mmHg and adding a right atrial pressure estimate of 5 mmHg [15]. Diastolic dysfunction was estimated by measurement of left atrial diameter [reference < 4.0 cm] and right atrial diameter (as assessed by short [< 4.1 cm] and long axis [< 5.0 cm]) as well as analysis of the E/A-ratio, defined as flow velocity of passive filling of the left ventricle divided by flow velocity of active filling due to atrial contraction [> 1].

### Statistics

All data are shown as mean ± standard deviation (confidence interval of 95%). Data were checked for normality by using the Kolmogorov-Smirnov test. Statistical comparison between patients and controls were assessed with the use of the student’s t-test. A p-value <0.05 was considered to be statistically significant. Spearman’s test was used for correlation analysis.

## Results

### Characteristics of patients and controls

46 patients with Martorell ulcer were retrieved from a retrospective data base. Twentytwo files concerned subjects who were already deceased, leaving a total of 24 eligible patients. One patient was excluded because of retrospectively revised diagnosis of peripheral arterial disease instead of Martorell ulcer. Eight patients refused to participate at the study. Fifteen patients were further evaluated for the presence of PH by echocardiography. One patient was excluded after analysis because systolic RV/RA gradient could not be measured due to absence of tricuspid regurgitation.

Patient data from 14 patients with Martorell ulcer were compared to data of the control group; details of patients’ characteristics are provided in Table 2. Briefly, 6 of 14 patients (42%) were male, the mean age was 77 years (range 70–89 years). Hypertension was present in all patients with Martorell ulcer (100%), type 2 diabetes mellitus was found in 5 of 14 patients (37%). 2 patients (14%) were still active smokers whereas 3 (22%) had stopped smoking (former smokers), 9 (64%) had never smoked. Creatinine levels and estimated glomerular filtration rate (GFR) as calculated according to MDRD (modified diet in renal disease formula [16]) were normal in 6 of 14 patients (42%) and in 12 of 28 controls (43%) [reference range 70 – 110 μmol/l]. Renal function was moderately reduced in 6 of 14 patients (CKD stage 3 in 42% patients) and in 14 of 28 controls (50%). 2 of 14 patients (16%) and 2 of 28 controls (7%) showed severe (CKD stage 4) reduction in renal function. Creatinine levels at time of echocardiography (109 ± 51 vs. 104 ± 42 μmol/l) and GFR according to MDRD (64 ± 37 vs. 59 ± 19 ml/min) did not reveal significant differences between patients and controls. Oral anticoagulation was established in 8 of 14 patients (50%) and in 8 of 28 controls (28%). Levels of antinuclear antibodies (ANA) were negative in 6 of 14 patients; elevations above 1:160, which is used as cut-off at the University Hospital Zurich, were observed in 2 patients (14%) without clinical signs of a systemic autoimmune disease. Since Martorell ulcer was diagnosed by tissue biopsy, no evidence for coumarin-induced skin necrosis or autoimmune disorders associated with the development of ulcerating lesions has been found.

**Table 2:** Comparison between patients and controls


Δ P systolic RV / RA – mmHg (± SD)	33.8 (± 16.9)	25.3 (± 6.5)	p = 0.023
Pulmonary hypertension (sPAP > 36 mmHg) – no. (%)	5 (36%)	2 (7%)	p = 0.031
Left ventricular ejection fraction – % (± SD)	62 (± 3)	58 (± 12)	ns
Left atrial diameter (ESD)– cm (± SD)	4.47 (± 1.05)	4.35 (± 0.80)	ns
Right atrial diameter (ESD) long axis – cm (± SD)	5.20 (± 1.04)	5.08 (± 0.83)	ns
Right atrial diameter (ESD) short axis – cm (± SD)	3.84 (± 0.80)	3.72 (± 0.75)	ns
E / A ratio – mean (± SD) *	0.90 (± 0.23)	0.91 (± 0.37)	ns
Significant valvular heart disease – no. (%) †	3 (21%)	5 (17%)	ns
Creatinine level – μmol/l (± SD)	109 (± 51)	104 (± 42)	ns
GFR estimated by MDRD – ml/min (± SD)	64 (± 37)	59 (± 19)	ns
Body Mass Index (BMI) – kg/m^2^ (± SD)	29.57 (± 7.08)	25.84 (± 5.07)	p = 0.036

* E / A ratio could not be assessed due to atrial fibrillation in 5 of 14 patients and in 8 of 28 controls.

† Mitral insufficiency in 1 of 14 patients and 3 of 28 controls; severe aortic stenosis in 2/14 and 2/28, respectively.

Body mass index (BMI, defined as body weight in kilograms divided per height in square meters) of patients with Martorell ulcer was significantly higher than in the control group (29.5 ±7.0 vs 25.8 ± 4.0 kg/m^2^).

### The RV/RA gradient is significantly elevated in patients with martorell ulcer

**Figure 3:**
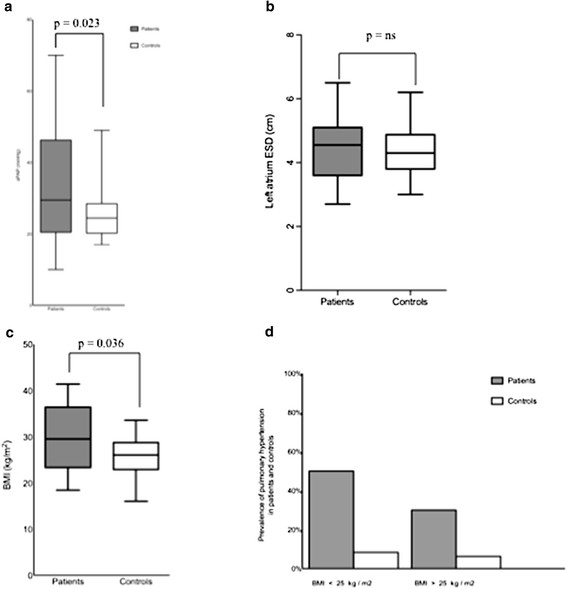
**Differences between patients and controls. a**) The RV/RA gradient is significantly higher in patients as compared to matched controls (p = 0.023); **b**) the end systolic diameter (ESD) of the left atrium is not significantly different between patients and controls; **c**) the BMI is higher in patients than in controls (p = 0.036); **d**) prevalence of PH in patients and controls with elevated BMI (cut-off 25 kg/m^2^). Data in a - c are plotted as median including upper and lower whisker.

As shown in Figure 3a, the RV/RA gradient systolic pulmonary arterial pressure (sPAP) in patients with Martorell ulcer (33.8 ± 16.9 mmHg) was significantly higher than the pressure measured in the control group (25.3 ± 6.5 mmHg, p = 0.023). The absolute difference between the means of the gradients of cases and controls was 8.5 mmHg. This difference was independent of left heart function, since all 14 patients showed a normal left ventricular ejection fraction (LVEF ≥ 50%, 62% ± 3). On the other hand, in 4 of 28 controls the LVEF was found to be impaired (58% ± 12); the difference observed between left ventricular function of patients and controls however was not significant. Dilatation of the left atrium (end-systolic-diameter (ESD) > 4 cm) is considered to be an important surrogate marker of diastolic dysfunction, which, in turn, might lead to pulmonary hypertension. Left atrial ESD exceeding 4.0 cm was found in 10 of 14 patients (71%) and in 16 of 28 controls (57%). The difference in left atrial ESD between patients (4.47 cm ± 1.05) and controls (4.35 cm ± 0.80) is illustrated in Figure 3b and was not statistically significant. Measurement of right atrial diameter assessed by long and short axis did not differ between patients and controls. Similarly, no significant differences were found in the E/A-ratio between the patient group and the control group.

### The prevalence of pulmonary hypertension in patients with martorell ulcer is significantly higher than in controls

When pulmonary hypertension was defined as sPAP >36 mmHg or as RV/RA gradient of >31 mmHg, respectively [15], the prevalence of pulmonary hypertension in patients with Martorell ulcer was 5 out of the 14 cases (36%). This figure was significantly higher than in the control group, in which only 2 out of 28 cases (7%) had an elevated RV/RA gradient (p = 0.031).

### The BMI in patients with martorell ulcer was higher than in controls

Of interest, the absolute difference of the BMI between patients and controls was 3.7 kg/m^2^; this difference did reach statistical significance (Figure 3c, p = 0.036). Figure 3d shows the prevalence of pulmonary hypertension in patients and controls with or without elevated BMI. 30% of patients with a BMI > 25 were found to have pulmonary hypertension as compared to 6.25% of obese controls. On the other hand, 50% of Martorell patients with a BMI < 25 were detected to have an elevated sPAP as compared to 8.3% of non-obese controls. For severity of pulmonary hypertension, no association between BMI and sPAP was found in patients or in the collective population of investigated patients and controls (data not shown).

## Discussion

In the present study, we investigated the prevalence of PH in subjects with Martorell ulcer and found that the sPAP as assessed by echocardiography is significantly elevated in patients as compared to matched hypertensive controls.

Martorell ulcer is caused by hypertensive changes in subcutaneous skin arterioles leading to reduced tissue perfusion; Martorell ulcer is, though underdiagnosed, an uncommon cause for leg ulcers [4]. PAH is another orphan disease defined by increased pulmonary arterial pressure due to remodelling of small pulmonary arteries. The list of associated conditions and risk factors predisposing for the development of PAH however has been growing constantly [13]. Moreover, when all conditions are considered that result in elevation of pulmonary pressure, PH is of emerging clinical relevance.

Histopathological changes observed in the peripheral arterial vessels within a Martorell ulcer include stenotic arteriosclerosis with calcification and hypertrophy of the vessels media, resulting in an increase of the wall to lumen ratio [Figure 1. Of interest, similar changes are seen in the remodeling of arteries described in the pulmonary vasculature of patients with several forms of chronic PH including hypertrophy and hyperplasia of smooth muscle fibers in the media of muscular arteries resulting in an increase of the cross sectional area [11,17].

In this case–control study, we found that patients with Martorell ulcer have significantly higher levels of sPAP compared to a matched control group. This difference has been assessed in a non-invasive manner by the use of transthoracic echocardiography. This method has been challenged by a recent study and was found to be inaccurate for diagnosing PH [18]; however, Doppler echocardiography is a validated and largely utilized modality to assess right ventricular function and is the currently established approach to screen patients for elevated pressure within the pulmonary circulation [19]. Of course, the method is limited for several reasons and does not permit direct conclusions on the type of PH. Right heart catheterization to confirm the diagnosis and evaluate the severity of PH was not considered as feasible approach within this distinct study population; study design and ethical proposal thus were based on echocardiographic assessment. Patients and controls did not show significant differences when compared for left ventricular function, volume of left and right atrium, E/A ratio or the presence of significant valvular heart disease.

However, the difference in BMI between patients and controls reached statistical significance. Together with the presence of other risk factors for a metabolic syndrome, such as age, systemic arterial hypertension and diabetes mellitus, these data suggest that diastolic heart failure is of major importance in the development of an elevated pulmonary arterial pressure – in both patients with Martorell leg ulcer and hypertensive controls. Of interest, however, more Martorell patients with a normal BMI developed pulmonary hypertension than obese patients. Whether the significantly increased prevalence of pulmonary hypertension in patients with Martorell leg ulcer might additionally be caused by other, pre-capillary factors remains, mainly due to the lack of invasive hemodynamic data and the absence of histopathological evidence for remodelling of pulmonary arterioles, hypothetically.

The main finding of this study, i.e. that the prevalence of an elevated pulmonary arterial pressure in patients with Martorell leg ulcer was significantly higher than in the control group, suggest that patients with Martorell ulcer appear to have an increased risk to develop PH, most probably due to diastolic heart failure, which can be prominent in patients with Martorell leg ulcer due to longstanding arterial hypertension. Other studies will have to investigate whether the presence of Martorell leg ulcer might additionally predispose for pre-capillary PH as it was shown similarly for hyperthyroidism [20], concomitant HIV infection [21] or smoking [22].

Our data further imply that hypertensive patients with leg ulcers who present with signs of right heart failure including dyspnea, peripheral edema, hypoxemia and reduced exercise capacity should undergo prompt echocardiographic evaluation to exclude or confirm the presence of an elevated sPAP. Since adequate treatment of hypertension does not prevent Martorell ulcer [4], it seems unlikely that the development of PH can be avoided by the control of systemic blood pressure. On the other hand, it remains unclear whether the specific therapy with vasodilatating agents (e. g. phosphodiesterase-5 inhibitors or endothelin receptor antagonists) might improve tissue perfusion and, thus, would enhance the wound healing of Martorell ulcer. However, patients with Martorell ulcer and concomitant PH could benefit from early diagnosis and onset of an adequate therapy.

Our study is limited, as mentioned above, by the fact that pulmonary arterial pressures were assessed non-invasively by echocardiography and not by right heart catheterization; moreover, the study lacks supporting biomarker data, and, finally, the case number is low, which is due to the fact that Martorell ulcer is a rare disease. Since this study was designed as observational and not as interventional study, the low case numbers should not severely affect our conclusions.

## Conclusion

Taken together, this is to our knowledge the first report showing a potential association between the development of PH and Martorell ulcer. Patients with Martorell ulcer might be at significant risk to develop PH. Our data emphasize that hypertensive patients with leg ulcers should promptly be screened for the presence of an elevated pulmonary arterial pressure when presenting with dyspnea, hypoxemia or reduced exercise capacity.

## Abbreviations

BMI, Body-Mass-Index (kg/m2); CKD, Chronic kidney disease; ESD, End systolic diameter (cm); GFR, Glomerular filtration rate (ml/min); LVEF, Left ventricular ejection fraction (%); MDRD, Modified diet in renal disease formula; n/a, Not applicable; PAH, Pulmonary arterial hypertension; PH, Pulmonary hypertension; RAD, Right atrial diameter; RV/RA, Right ventricular – right atrial pressure gradient (mmHg); sPAP, Systolic pulmonary arterial pressure (mmHg).

## Competing interests

The authors declare that they have no competing interests.

## Authors’ contributions

LGvB, MF, RS, SU and LCH contributed to the design of the study and data collection, performed statistical analysis and interpreted data, FCT performed transthoracic Doppler echocardiography, JH contributed to data collection. GN and MB reviewed drafts of the manuscript. All authors read and approved the final manuscript.
